# Well-Being and Romantic Relationships: A Systematic Review in Adolescence and Emerging Adulthood

**DOI:** 10.3390/ijerph16132415

**Published:** 2019-07-07

**Authors:** Mercedes Gómez-López, Carmen Viejo, Rosario Ortega-Ruiz

**Affiliations:** Department of Psychology, Universidad de Córdoba (Spain), 14004 Córdoba, Spain

**Keywords:** well-being, psychosocial adjustment, emotional adjustment, optimal functioning, romantic well-being, positive psychology, PRISMA protocol

## Abstract

Adolescence and emerging adulthood are both stages in which romantic relationships play a key role in development and can be a source of both well-being and negative outcomes. However, the limited number of studies prior to adulthood, along with the multiplicity of variables involved in the romantic context and the considerable ambiguity surrounding the construct of well-being, make it difficult to reach conclusions about the relationship between the two phenomena. This systematic review synthesizes the results produced into this topic over the last three decades. A total of 112 studies were included, following the Preferred Reporting Items for Systematic Review and Meta-Analysis Protocols (PRISMA-P) guidelines. On the one hand, these works revealed the terminological heterogeneity in research on well-being and the way the absence of symptoms of illness are commonly used to measure it, while on the other hand, they also showed that romantic relationships can be an important source of well-being for both adolescents and emerging adults. The findings underline the importance of providing a better definition of well-being, as well as to attribute greater value to the significance of romantic relationships. Devoting greater empirical, educational, and community efforts to romantic development in the stages leading up to adulthood are considered necessary actions in promoting the well-being of young people.

## 1. Introduction

Since World War II, most conceptualizations of health have been focused on the absence of illness and disability [1]. Psychology was concentrated on repairing damage within a disease model of human functioning [2], paying almost exclusive attention to pathology and neglecting the study of the positive features that make life worth living [2]. It is currently known that the absence of pathology does not necessarily correlate with positive dimensions of health and well-being [3,4], and psychologists have begun to admit well-being as a relevant aim of study, as well as the factors that contribute to its encouragement [5]. Positive psychology was recently established as a new perspective specifically addressing the study of well-being, quality of life, strengths, and resources [2,6]. Within this framework, diverse approaches have emerged. In a general sense, well-being can be understood as optimal psychological functioning and experience [7]. More specifically, some theorists have defined it as a state characterized by a high degree of satisfaction with life and the experience of high levels of positive affect [8], while others have focused on the notion of a process of fulfilling human potentials, capacities, and virtues [7]. Despite this systematization of the theory, the diversity of terminology found in the different studies has led to a certain degree of controversy. Although, admittedly, this situation has contributed to a productive scientific debate, it has also led to considerable ambiguity and theoretical and methodological confusion. On the other hand, these approaches represent mainly personal evaluations of what well-being means, and they deal only fleetingly with the social dimension of the individuals involved. In this sense, it has been previously established that the desire for interpersonal attachment (the need to belong) is a fundamental human motivation [9], especially when it refers to romantic relationships. So important is relatedness that some theorists have defined it as a basic human need, essential for well-being [9,10,11,12]. For example, in their 2002 study, Diener and Seligman examined extremely happy people to determine necessary conditions for entering this group [13]. They found that good and strong personal relationships were ubiquitous in these people. Nevertheless, the topic of relationships is complex and close relationships are multifaceted, justifying with this a study of specificity, in terms of the aspects of relationships that can promote well-being [7]. 

### 1.1. Romantic Relationships and Well-Being in Adolescence and Emerging Adulthood

From an evolutionary point of view, adolescence and emerging adulthood (the periods which span the second and third decades of life [14,15]) have been described as being vitally important in terms of the development of romantic relationships [16,17,18]. Defined as “mutually acknowledged ongoing voluntary interactions” [18,19], these relationships, unlike others such as friendships, are characterized by a particular intensity, specific expressions of affection, and initiation in erotic sexual encounters [19]. Previous studies have shown that these experiences are frequent during adolescence and tend to consolidate over time [20,21], representing an important context for learning and training for future intimate relationships [14]. By middle adolescence, most boys and girls have been involved in at least one romantic relationship [21], providing them with a scenario characterized by greater intimacy, support, and importance as their age advances [22,23]. As adolescents approach emerging adulthood, the time they devote to their romantic partners increases [24,25], and they use these relationships to look for company, emotional security, intimacy, and the feeling of love they provide, until they reach a stage when they are ready to take decisions over questions of long-term commitment, such as cohabitation and marriage [26,27]. According to the developmental task theory, during adolescence, romantic involvement is an emerging developmental task, which will eventually become a salient developmental task in adulthood [28].

Romantic relationships and experiences are important sources of emotional bonding and contribute to the development of a positive self-concept and greater social integration [29,30]. The successful establishment and maintenance of romantic relationships can have important repercussions in later stages of life [15], and has been described to contribute to people’s mental and physical health and, therefore, to their well-being [31]. From this perspective, romantic relationships, when sustained over time, constitute a transformation of the attachment bond. The quality of the relationship, the history of the shared experiences, the sense of attachment, and the beliefs which arise from the whole experience have all been recognized as modulating the well-being of the partners [32,33,34,35,36,37,38,39,40]. Despite the fact that the wide range of aspects mentioned in the research makes it difficult to establish how direct an effect these relationships have on well-being, there is a broad consensus in the literature that love is one of the strengths most closely linked to personal happiness [41,42], and is associated with higher rates of self-esteem, safety, satisfaction with life, positive affect, and achievement of personal and relational goals [43,44,45,46]. However, romantic relationships have also been associated with negative outcomes, especially during adolescence. Thus, studies have suggested that romantic involvement may be related to the presence of different forms of violence [47,48,49,50], experiencing internalizing symptoms such as depression or anxiety (e.g., [37,51,52]), poorer psychosocial functioning [53], or delinquency [54].

### 1.2. The Present Study

Following these considerations, the empirical evidence suggests the important role that romantic relationships can play in people’s well-being, however, the number of studies focusing on stages prior to adulthood remain relatively limited, consequently not providing clarifying results. Moreover, the wide range of intervening variables in the romantic context and the relative ambiguity of the concept of well-being make it difficult to draw conclusions. Therefore, a work of synthesis is required to gather together the accumulated empirical knowledge and facilitate an understanding of the findings made so far in relation to the association between well-being and romantic relationships in adolescence and emerging adulthood. To do this, the general aim of this study was to carry out an exhaustive review of the existing literature in order to delve deeper into this topic. In particular, a specific aim was established: To identify the variables of romantic relationships that studies have associated with the well-being of young people.

## 2. Materials and Methods 

### 2.1. Literature Search and Quality Assurance

A structured search was carried out between July and September 2017 in the following databases of high-quality standards, which include peer-reviewed studies: Scopus, Web of Science, PsycINFO and Scielo. The Preferred Reporting Items for Systematic Review and Meta-Analysis Protocols (PRISMA-P) Declaration was applied [55], following its protocol for the planning, preparation, and publication of systematic reviews and meta-analyses [56]. The search terms used included keywords in Spanish and English which were considered to be indicators of well-being (bienestar*, well-being*, wellbeing*, “wellbeing”, felicidad*, happiness*, “fortalezas psicológicas”, “psychological strengths”, florecimiento*, flourishing*, “desarrollo positivo”, “positive development”) and keywords linked to romantic relationships (dating*, “relaciones sentimentales”, “sentimental relationships”, “relaciones románticas”, “romantic relationships”, cortejo*, courtship*, “relaciones íntimas”, “intimate relationships”). In order to achieve a comprehensive overview of the state of research in this field, the search did not include any specific terms (e.g., psychological well-being, subjective well-being, hedonia, eudaimonia, hooking up, friends with benefits, etc.).

### 2.2. Inclusion/Exclusion Criteria

The inclusion criteria were established following the PICOS (acronym for Participants, Interventions, Comparisons, Outcomes and Study design) format [57]:

- Type of participants: Adolescents and emerging adults of both sexes, ranging in age from 13 to 29 years old, or those whose average age is included in that range, with no known mental disorders, and those of any origin or nationality.
- Type of studies: Empirical studies written in English or Spanish and published in peer-reviewed journals.
- Type of outcome measurements: In a first stage, studies were included which made explicit reference to the search descriptors in the title, summary, and/or keywords. In a second stage, studies were included with specific analyses of the link between romantic relationships and any of the previous indicators.
- Type of designs: Quantitative and qualitative.

Additional exclusion criteria included theoretical studies, doctoral theses, systematic reviews, meta-analyses, book chapters, reports from conferences or symposia, letters to the editor, minutes of meetings or informative notes, and studies in which the authors did not provide information about the participants’ age.

### 2.3. Data Coding and Extraction

**Figure 1:**
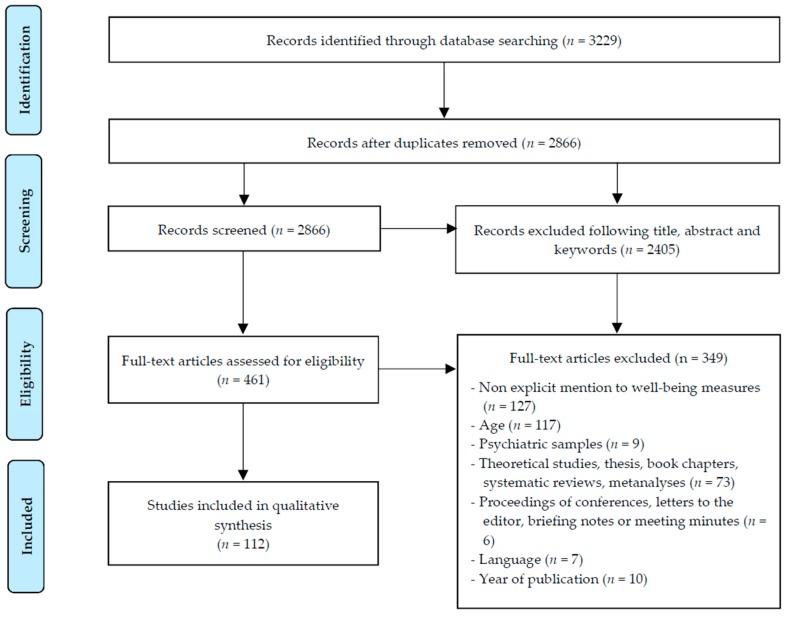
Preferred Reporting Items for Systematic Review and Meta-Analysis (PRISMA) flow diagram.

Three matrices of documentary records were created specifically for this work. In the first, quantitative data on the search results were collected for each database consulted and each of the descriptors used. In the second, information was gathered from each selected or unselected study (e.g., title, author/s, year of publication, sample size, age of participants, study objectives, methodology, or reason for exclusion, where appropriate). The third recorded the well-being measures and the specific variables of the romantic context analyzed by the studies. The selection of studies was performed in different stages [58] (Figure 1). The identification stage was limited to articles published in English and Spanish between 1990 and 2017 (inclusive). This first phase yielded a total of 3229 studies. In the screening stage, the duplicates were discarded, which left a total of 2866 studies. Next, two reviewers selected the studies whose title, summary, or keywords contained any of the search descriptors used, which produced a total of 461 eligible studies and a total of 2405 rejected studies. In the eligibility stage, all the reviewers independently assessed the full text of the potential studies to be included, initially reaching a level of agreement of over 90% and resolving any discrepancies through a process of discussion and consensus. In the included stage, the three reviewers jointly agreed on the full sample of studies, resulting in a total of 112 studies. The software packages Mendeley version 1.17.12 (Elsevier Inc., New York, NY, USA) and SPSS version 22 (IBM Corp., Armonk, NY, USA) were used to carry out the process of coding and obtaining the results. 

## 3. Results

### 3.1. Characteristics of the Included Studies

This work has reviewed nearly three decades of research (1990–2017) on well-being and romantic relationships during adolescence and emerging adulthood. Of the 112 studies included (see Table 1), 9% were published in the 1990s, 27% in the first decade of this century, and 64% were published since 2010. The total number of participants was 278,871, with the amount of participants ranging from 30 in some studies [59,60] to 81,247 participants in another [47]. The general age range was from 12 to 70 years, with the average age never surpassing 29 years in any of the studies. Overall, 83% of the studies (n = 93) were directed at emerging adulthood, while 17% (n = 19) focused on adolescence. Regarding the well-being measures observed, the studies analyzed used as many as 142 different variables, of which the most commonly employed were life satisfaction (35.3%), depression (25%), affect (positive and negative, 22.8%), self-esteem (17.6%), relationship satisfaction (15.4%), anxiety (11%), happiness (8.1%) and stress (5.9%).

### 3.2. Variables of Romantic Relationships Related to Well-Being in Adolescence and Emerging Adulthood

Achieving the specific aim of this study involved reviewing the variables of romantic relationships which have been associated with well-being during adolescence and emerging adulthood. These variables were sorted into two categories: First, the label “relational variables”, where studies analyzing characteristics of romantic relationships and the processes that take place within them were grouped. Secondly, the label “personal variables”, which gathered the studies that examined individual variables involved in establishing, forming, and/or developing romantic relationships (see Table 2).

A total of 87 studies analyzed the association between romantic relationships and well-being based on relational variables. Relationship status, relationship quality, and relationship history and experiences were the variables most commonly focused on in the studies. In general, particularly during emerging adulthood, participants involved in a romantic relationship showed higher levels of well-being than those who were single. More specifically, it was suggested that staying single, either voluntarily or involuntarily, and remaining so in order to avoid the negative consequences of relationships (avoidance goals) was not associated with well-being, with the best predictor being satisfaction with that status. Particular aspects of relationship status, such as the stability of the relationship or the experience of splitting up, have been widely studied. Studies that equated commitment to romantic status suggested that a higher level of commitment or stability in the relationship (marriage vs. cohabitants, non-marital relationships, casual relationships, etc.) leads to a greater well-being. In this regard, a specific case analyzed was hook-up experiences. These expressions of sexuality, outside the context of a committed relationship, were only negatively associated with well-being in one study. Similarly, the experiences of separation or divorce have been identified with increased well-being if these events were evaluated positively, if the quality of the relationship was poor, or if a new relationship started shortly after the separation. 

Along similar lines, the studies also evaluated the role of well-being in relationship quality, with relationship satisfaction, commitment and intimacy being the most common indicators. Throughout the periods of adolescence and emerging adulthood, high levels of quality in the relationship were positively associated with well-being, while, similarly, low levels of quality were linked to negative effects. In cases of transgression, the quality of the relationship was also identified as a mediator between forgiveness and the well-being of the transgressor. Close to the findings regarding relationship quality are those associated with relationship history and experiences. The studies in this line showed that reporting and remembering a large number of positive experiences, such as shared laughter, being at a formal or positive relational turning point, or expressing gratitude towards the partner, were all positively associated with well-being, while negative experiences, such as arguments, transgressions, power imbalance, or violence, were associated with a decrease in well-being levels. 

When considered independently and not as indicators of the relationship quality, rates of commitment and intimacy between partners have also been identified as variables which can influence well-being: High levels of commitment to the relationship and intimacy between romantic partners were positively associated, where low levels of commitment showed an inverse relationship. Likewise, romantic attachment can also have important implications. The studies indicate that a secure romantic attachment would be most beneficial, while avoidant and anxious attachment have been suggested as reliable predictors of low levels of well-being.

Communication and conflict resolution between partners have both been identified as variables with a significant effect on well-being. On one hand, the disclosure of sexual problems and receiving positive body feedback from the partner were both positively associated with well-being, while on the other hand, showing high levels of positive affect in conflict situations was found to be a good predictor of relationship stability and satisfaction. Likewise, self-compassion and dyadic empathy (empathy specifically expressed towards the romantic partner) were variables found to have a positive effect, where more self-compassionate individuals were more likely to resolve interpersonal conflicts by balancing their needs to their partner’s needs, feeling more authentic and less emotionally turmoiled. Similarly, high levels of empathy in couples in the transition to parenthood led to improved levels of well-being in the partners. 

Variables concerning need fulfillment and achieving relational and personal goals have also been identified as related to well-being. A partner’s support to personal needs of autonomy, competence, and relatedness (Self-Determination Theory [8]), or the maintenance of relational behaviors driven by self-determined motives, were positively associated with well-being. Similar results were found in relation to the effects of the achievement of the ideal self and the congruence of the goals between partners. According to the studies, romantic partners can significantly influence what we become, having important implications for well-being, as well as the pursuit and involvement in activities aimed to achieve shared goals. 

In the studies conducted during adolescence, violence occurring within the relationship (dating violence) in either form, both as a victim and as a perpetrator, has emerged as a highly significant negative variable for well-being, being associated to symptoms of anxiety, depression, stress, and low levels of self-esteem and life satisfaction, among other symptoms. Other relational variables associated with well-being during adolescence were the maintenance of same-sex relationships and interracial relationships, as well as sexuality. The negative impact caused by expected rejection due to sexual orientation was buffered by involvement in same-sex relationships, as well as improved self-esteem and decreased levels of internalized homophobia. Conversely, interracial daters were found to be more likely to suffer from depression and anxiety, as well as to perceive less support from parents and family, compared to same-race daters and non-daters. In relation to sexuality, results showed that the influence of sexual activity in depression was differentially associated with romantic status, where sexual relations associated with greater depressive symptoms corresponded to those that occurred outside the context of a romantic relationship. On the other hand, longitudinal data associated high sexual health with higher levels of well-being in adolescent girls, using indicators such as physical, mental/emotional, and social health.

To a lesser extent, the studies reviewed addressed aspects related to relationship dynamics and their association with well-being. Research into emotional interdependence (i.e., partners’ emotions being linked to each other across time), shared relationship efficacy (i.e., partners’ shared expectations about the joint ability to maintain satisfactorily the relationship), partner-specific perfectionism concerns, or the effect of relationships at the neurological level has rarely been contrasted with other studies. Despite this, the first two aspects were established as characteristics of healthy relationships with a positive influence on well-being, however, concerns about perfectionistic demands of the partner (perceived partner’s expectations about one’s own mistakes, self-criticism, and socially prescribed perfection) generated and evoked socially negative behaviors, which in turn had a deleterious effect on negative affect and life satisfaction. 

Regarding the personal variables, a total of 25 works studied their relationship with well-being. Here, the variable which received the most attention was the belief system. It has been shown that, during adolescence, the imbalance between romantic expectations and reality (romantic relationship inauthenticity) is associated with a greater risk of depression and suicidal behavior, while the Sense of Coherence (SOC), a dispositional orientation or a coping resource which reflects a person’s capacity to respond to stressful situations and life events, is linked with greater life satisfaction. In emerging adulthood, relationship expectations and beliefs were also suggested as factors influencing well-being. The congruence between previous expectations and reality, or between the ideal and the real romantic relationship, has been identified as a good indicator of well-being. There is no general consensus over the results for other kinds of beliefs, such as positive illusions (idealizing the partner), marriage myths, or benevolent sexism, although a number of studies have addressed them. The tendency is that the first two seem to be beneficial for well-being, while the latter showed a negative association.

In addition, certain types of behaviors, which may be induced by beliefs, also seem to impact well-being. On the one hand, behaviors which diminished satisfaction with the relationship, such as sexual compliance (voluntary maintenance of unwanted sex with a partner), have been negatively associated with well-being. On the other hand, behaviors linked to self-knowledge or positive management of the relationship, such as making attributions and reasoning about the mental state of others (i.e., theory of mind), self-control, authenticity (acting in a way which is congruent with one’s own values, beliefs, and needs), or the use of effective coping strategies in stressful events, were positively associated with well-being. In this sense, focusing on the problem or perceiving the situation as controllable had positive effects on well-being in cases of abuse or violence within the relationship. In less serious cases, maintaining an implicitly positive attitude towards the partner and mindfulness obtained similar results.

Regarding cognitive, emotional, and behavioral motivation, self-forgiveness or approach and avoidance motives were revealed as indicators of well-being. According to the analyzed studies, forgiving the partner or forgiving oneself, regarding harmful relationships events, was positively related to well-being. Moreover, engaging sexually with the partner increased well-being, but only when these motives were based on approximation towards positive consequences (e.g., happiness of the partner or promoting the intimacy of the relationship) and not on the avoidance of negative consequences. Similar results were found in relation to sacrifice. Self-sacrificing aimed at achieving beneficial goals, that is, pro-social behavior which gives priority to benefits to the relationship over personal benefit, has also been positively related to well-being. Conversely, emotional suppression, limiting one’s partner’s attention towards attractive alternatives, or the pursuit of traditionally masculine roles (e.g., success, competition, or power) negatively affected the partner. Finally, the level of romantic competence and other skills that promote the establishment and successful maintenance of relationships, such as perceived self-efficacy, or the ability to control relational anxiety, have been strongly linked to positive results, as well as a greater ability to make better decisions and feel more confident and satisfied with the relationship.

## 4. Discussion 

The main aim of this study has been to carry out a systematic review of the scientific literature on the association between romantic relationships and well-being during adolescence and emerging adulthood, focusing on identifying the specific variables associated with well-being in the romantic context.

In the first place, it is important to stress that well-being has been historically been measured in many different ways. The great number of variables observed have produced a potential problem of construct validity. It seems clear that the multiple conceptual and operational definitions used in the empirical studies on well-being hinder rather than help when it comes to defining this construct [151,152]. It is therefore important to continue trying to bring clarity to a field which is still in evolution, with previous works and new approaches still trying to be integrated [6]. Although this has its positive side, it also highlights a greater need for improving the theoretical approaches, making them more global in terms of personality and also more precise in terms of the relationship between personality traits and relational styles in romantic processes. Another aspect which may contribute to the lack of clarity in the concept of well-being is the continued use of symptoms of mental illness as an indicator. While it is true that not all of the studies reviewed used this clinical approach, but rather adopted models from positive psychology (e.g., [22,33,44,85,97,98]), there is still a prevalent tendency to conceptualize well-being in terms of the absence of disease or clinical symptoms, rather than providing a positive approximation to the concept. This is quite surprising, especially considering that it has previously been established that health and mental illness work in a relatively independent manner [153], and that the factors which make either reduce do not necessarily cause the other to increase [154]. The concept of mental health proposed by positive psychology is therefore of particular relevance here, although the definition used (the existence of a high level of well-being and the absence of mental illness) [153,155] suggests the need to develop a methodologically diverse theory which would include the full spectrum of well-being [151] and to adopt a theoretical approach according to the concepts measured, which, as of yet, none is present in the reviewed works.

In the second place, it is clear that the scientific literature stresses the importance of romantic relationships during adolescence and emerging adulthood [18,156,157], however, the small number of studies which have focused specifically on these stages show that there is a need to provide a specific psycho-evolutionary focus. Based on the works reviewed, it can be stated that romantic relationships are significantly associated with well-being in adolescents, although a number of different personal and relational variables can be understood as risk factors. A low SOC, a lack of authenticity, or the presence of violence in relationships [37,48,49,59,78,83,108,122,142], are harmful to adolescents, all of which can be explained from different perspectives. On the one hand, according to the normative trajectory model [158], early romantic experiences can compromise the well-being of adolescents when dealing with non-normative development events. On the other hand, the stress and coping model [159] postulates that romantic relationships are intrinsically challenging, requiring skills and resources that adolescents may not have. Following studies like those of [160] and [161], it is also plausible to pose the counter-argument that high levels of well-being could act as a protective factor, promoting healthy behaviors. Research with adult populations has already established this association and suggests that people with high levels of life satisfaction are more involved in intimate activities and relationships and have better relationships [13,85,162]. The association between well-being and romantic experiences during adolescence seems, therefore, to operate under a bidirectional pattern of influence, revealing with this the existence of a more complex relationship between both processes. Besides this, it is also especially important to remember that the romantic development of adolescents does not take place in a social vacuum, so it is vitally important for the well-being of adolescents to have social contexts which provide support and emotional understanding as they face the demands and challenges that this new evolutionary task lays on them [163].

Just like in adolescence, involvement in romantic relationships can be a significant source of well-being in emerging adulthood. The research reviewed suggests that young adults who have romantic relationships are happier, feel more satisfied with their lives, have fewer problems with mental and physical illness, show greater positive affect, and have better levels of self-esteem than single people. However, as noted above, the phenomenon of romantic relationships is complex and multifaceted and is associated with both relational and personal factors, and not only with their presence or absence. The relationship quality, the satisfaction of the needs of autonomy, competence and relatedness and a secure attachment with the partner have been indicated as strong indicators of well-being [7,164]. In addition, variables such as high levels of commitment, intimacy, communication, providing support to achieve personal and relational goals, good conflict management, approach motives (in contrast to avoidance), authenticity, or having strategies for coping with stressful situations, are also associated with good results, as confirmed by other studies [165,166]. Finally, personal skills and having the competence to maintain healthy and satisfying relationships are important factors which, according to some studies, can reduce symptoms of depression and anxiety, increase satisfaction with the relationship, the development of a secure attachment, and foster better decision-making. For this reason, romantic relationships based on principles of mental and emotional health and romantic competence [23,167] are considered to be among the prime sources of well-being during emerging adulthood.

## 5. Conclusions

Based on these results, one of the main conclusions from this study is the invaluable role which romantic relationships play in well-being during adolescence and emerging adulthood. As a result, this work supports their consideration as developmental assets [14]. However, the numerous benefits which are associated with them call for certain parameters to be agreed on. A relationship which is beneficial for well-being would, in general terms, have high-quality levels, through which the partners can develop their potential, achieve personal and shared goals, and maintain a secure attachment. To achieve this, people must achieve certain cognitive, emotional, and behavioral skills. It is proposed that these principles be integrated into a more parsimonious analysis, which could aid our understanding of positive romantic relationships. From this viewpoint, this study proposes romantic well-being as a new term of analysis and suggests that in future research it can be understood and evaluated as a specific category. One of the main strengths of this work, therefore, is the initial approach of a new theoretical model, termed the multidimensional model of romantic well-being, whose dimensions correspond to the particular factors which, according to the research, play an especially important role in achieving positive results, namely relationship quality, need fulfillment, the achievement of personal and relational goals, romantic attachment, and the development of individual skills. 

Regarding the empirical approach to well-being, the main conclusion here is that it is necessary to understand the concept of well-being in of itself, without continually referring to a disease or symptom. This distorts the construct and prevents from relating it to dimensions which are also complex and rather diverse, such as those involved in the psycho-evolutionary task of adolescents maintaining a romantic relationship. Therefore, further research is required to establish a common, shared, and reliable theoretical and methodological framework for well-being, also allowing the ability to address the scientific study of romantic relationships in stages prior to adulthood, especially during adolescence. It is essential to adopt educational, clinical, and community models which focus on the need to promote positive, healthy, and satisfactory relationships, as well as raising awareness of this need among all professionals responsible for people’s health.

## Figures and Tables

**Table 1:** Characteristics and main findings of the included studies.

Reference	*N*	Age Range/School Level, Mean Age (SD)	Well-Being Measures	Main Findings
[47]	81,247	9th–12th grades, NR (NR)	Mood, stress, sadness, worry, hopelessness, and life satisfaction.	Date violence and rape are associated with higher rates of suicidal thoughts and attempts, and lower scores of well-being.
[61] Study 4	119	NR, 21 (NR)	Level of happiness.	Explicit expressions of commitment were positively related with happiness.
[61] Study 5	73	18–57, 28 (NR)	Positive emotion.	
[44]	553	20–30, 23.42 (3.27)	Happiness, interest in life, life satisfaction, positive affect; autonomy, environmental mastery, personal growth, positive relationships, purpose in life, self-acceptance (Ryff’s model of psychological well-being); social acceptance, social actualization, social contribution, social coherence and social integration (Keyes’ model of social well-being).	Single individuals reported lower well-being than partnered individuals.
[62]	151	20–26, 22.48 (2.01)	Happiness, interest in life, life satisfaction, positive affect; autonomy, environmental mastery, personal growth, positive relationships, purpose in life, self-acceptance (Ryff’s model of psychological well-being); social acceptance, social actualization, social contribution, social coherence and social integration (Keyes’ model of social well-being).	Voluntarily and involuntarily single young adults differed neither regarding well-being.
[63] Step 2	185	20–32, 22.59 (3.23)	Happiness, interest in life and life satisfaction, positive affect; autonomy, environmental mastery, personal growth, positive relationships, purpose in life, self-acceptance (Ryff’s model of psychological well-being); social acceptance, social actualization, social contribution, social coherence and social integration (Keyes’ model of social well-being).	Relationship status satisfaction was found to be a good predictor of life satisfaction and well-being.
[64]	67 couples	19–56, 25.16 (6.33)	Daily relationship satisfaction and daily relationship connection.	Gratitude from interactions predicted increases in relationship connection and satisfaction.
[65]	53 couples	23–53, 29 (NR)	Life satisfaction and relationship satisfaction.	Partner responsiveness to gratitude expressions was related with greater well-being.
[66] Study 2	77 couples	Males: NR, 19.90 (2.31) Females: NR, 19.30 (1.20)	Life satisfaction.	As efficacy expectations shared between partners increased, the degree of their life satisfaction also increased.
[67]	63 couples	NR, 21.60 (2.79)	Happiness toward the relationship, closeness and intimacy.	Relationship identification predicted association between partner transgressions and well-being.
[68]	309	16–24, NR (NR)	Life satisfaction, positive/negative affect, optimism and self-esteem.	Minority stress components were negatively related to well-being, however, the impact of “expected rejection” on well-being was buffered for those involved in a romantic relationship.
[69] Study 1	76	NR, 22.43 (5.11)	Sadness.	Relationship maintenance behaviors were negatively associated with sadness when intimates subsequently reported high relationship satisfaction, but positively associated when intimates subsequently reported low relationship satisfaction.
[69] Study 3	135	NR, 26.90 (4.57)	Depressive mood.	Tendency to compromise during problem-solving was associated with less depressive mood among people who subsequently were more satisfied with their relationship.
[70]	139	17–51, 28.4 (6.9)	Mood, capacity to enjoy and relax, and capacity for social contact.	Implicit attitudes towards partners correlated significantly with explicit attitudes, secure attachment, and well-being.
[71] Study 1	89	18–23, 19.3 (NR)	Relationship satisfaction.	Higher trait mindfulness predicted higher relationship satisfaction and greater capacities to respond constructively to relationship stress.
[71] Study 2	60 couples	18–25, 20.05 (NR)	Relationship satisfaction.	Trait mindfulness was found to predict lower emotional stress responses and positive pre- and post-conflict change in perception of the relationship.
[72]	350	15–19, 17 (1.27)	Self-esteem, internalized homophobia, depression, and anxiety.	Involvement in same-sex relationships was associated with self-esteem and internalized homophobia, where the timing and sequence of both had different effects on males and females.
[73]	52	Males: NR, 22.29 (3.13) Females: NR, 21.29 (2.40)	Relationship satisfaction.	Couples who reminisced about events involving shared laugher reported higher relationship satisfaction.
[74]	1584	18–25, 20.19 (NR)	Mental health problems, physical health problems, and overweight/obesity.	Individuals in committed relationships experienced fewer mental health problems and were less likely to be overweight/obese.
[75] Study 1	77	18–39, 20 (3.19)	Positive and negative affect.	People who were single for a shorter period of time were more likely to report higher levels of well-being.
[75] Study 2	236	18–49, 21.71 (5.63)	Positive and negative affect.	People who started a new relationship quickly had higher well-being compared to those who waited longer to begin their subsequent relationship.
[76]	62 couples	NR, 19.47 (1.53)	Autonomy, environmental mastery, personal growth, positive relationships, purpose in life, self-acceptance (Ryff’s model of psychological well-being); life satisfaction and affect-balance.	Authenticity was related to engaging in healthy relationship behaviors, which in turn predicted positive relationship outcomes and greater well-being.
[77] Study 1	202	NR, 18.81 (2.09)	Self-esteem, affect-balance, vitality, and life satisfaction.	Self-determined sexual motives positively predicted well-being.
[77] Study 2	147	NR, 19.10 (1.76)	Self-esteem and life satisfaction.	Self-determined sexual motivation, sexual need satisfaction, well-being, and relational quality were positively intercorrelated.
[77] Study 3	44 couples	NR, 19.10 (1.76)	Self-esteem and life satisfaction.	Men’s and women’s self-determined sexual motivation predicted their own well-being, and men’s self-determined sexual motivation also predicted women’s well-being.
[78]	12,203	12–19, 15 (NR)	Depression, conflicts, loneliness, anxiety, mental clarity, irritation, school performance, distrust, and to find it difficult to handle problems.	Adolescents in violent relationships are more likely to experience negative well-being outcomes.
[48]	190	13–19, 15.9 (1.29)	Anxiety, depression, self-esteem, life satisfaction, and traumatic symptomology (stress and dissociation).	Increasing levels of dating violence were related to higher levels of post-traumatic stress and dissociation in girls. Victimization was related to higher levels of anxiety, depression, and post-traumatic stress in boys.
[79] Study 1	112	19–54, 22.04 (4.37)	Depressed mood, self-esteem, life satisfaction, fatigue, perceived acceptance by one’s partner, relatedness, and relationship satisfaction.	Emotional suppression was related to a greater depressive mood, greater fatigue, lower self-esteem, lower life satisfaction, and less relationship satisfaction.
[80]	2214	17–25, 19.36 (1.51)	Difficulties in interpersonal relations, difficulties in social roles, and symptom distress.	Distress symptoms, difficulties in interpersonal relations, and difficulties in social roles were predicted by secure attachments to romantic relationships, among others.
[32]	99	18–33, 23.12 (2.43)	Anxiety and depression.	Endorsement of marriage myths predicted positive experiences, whereas benevolent sexism predicted negative experiences.
[81]	1040	18–24, 21.02 (1.92)	Depressive symptoms, anxiety symptoms, self-esteem, and sense of personal competency.	Negative appraisals of breakups were associated with lower well-being. Positive appraisals were associated with greater anxiety symptoms, self-esteem, and a sense of personal competency.
[45]	2273	14–19, NR (NR)	Positive self-view, depressive feelings, alienation and expectation of success in school, relationships, work, and health.	Being in a dating relationship was associated with less alienation, more positive views of the self, and higher general expectations for success. Among sexually active youth, daters had lower levels of depression than non-daters.
[82]	12,841	18–32, NR (NR)	Earnings, high relative income, and stability of employment histories.	Well-being had a weaker association with cohabitation than with marriage.
[83]	5414	14–18+, NR (NR)	Quality of life (stress, depression, problems with emotions, physical health, and suicide ideation and attempts) and life satisfaction.	Among girls, dating violence victimization was associated with poor health-related quality of life and suicidal ideation or attempts. Among boys, dating violence perpetration was associated with a poor health-related quality of life and suicide attempts, and lower scores of life satisfaction.
[43] Study 1	102	18–25, 20.9 (1.7)	Depression and anxiety symptoms, relationship satisfaction, romantic attachment security, and relationship decision making.	Romantic competence was associated with greater security, healthier decision making, greater satisfaction, and fewer internalizing symptoms.
[43] Study 2	187	NR, 19.65 (3.51)	Depression and anxiety symptoms, relationship satisfaction, and romantic attachment security.	
[43] Study 3	89 couples	Males: NR, 20.65 (1.82) Females: NR, 20.16 (1.63)	Relationship satisfaction, romantic attachment security, and relationship decision making.	
[84]	102 couples	NR, 25.40 (5.08)	Autonomy, environmental mastery, personal growth, positive relationships, purpose in life, and self-acceptance (Ryff’s model of psychological well-being)	Touch was associated with enhanced affect in the partner and with intimacy and positive affect in the actor. Participants who were touched more often during the diary study week reported better well-being 6 months later.
[85] Study 1	221	19–28, 22.49 (4.65)	Global happiness.	Romantic relationship quality was positively related to happiness.
[85] Study 2	187	18–29, 22.02 (3.02)	Life satisfaction and positive and negative affect.	
[22]	311	18–28, 22.75 (4.74)	Life satisfaction and positive and negative affect.	Romantic relationship quality and conflict were predictors of happiness.
[31] Study 1	43	Undergraduate	Relationship satisfaction and commitment.	Limiting people’s attention to attractive alternatives reduced relationship satisfaction and commitment and increased positive attitudes toward infidelity.
[86]	125	15–23, NR (NR)	Depression, anxiety, physical symptomology, perceived stress, self-esteem, mastery, and self-efficacy.	Sexual-minority youths had comparable self-esteem, mastery, and perceived stress as did heterosexuals, but greater negative affect.
[13]	222	College students	Life satisfaction and affect balance.	Well-being was positively associated with good-quality relationships.
[59]	15 couples	18–35, 24.9 (4.3)	Autonomy, competency, self-esteem, general life satisfaction, clarity/certain in life, social satisfaction, and social support.	Perceived understanding among romantic partners was positively associated with well-being.
[87]	63 (time 1)	NR, 19.10 (NR) -time 1-	Life satisfaction, emotional well-being, self-esteem, loneliness, relationship satisfaction, and relationship breakup.	The Michelangelo phenomenon was positively associated with well-being.
[88] Study 1	53 couples (time 1)	NR, 19.94 (NR) -time 1-	Intimacy, agreement, effective problem solving, and shared activities.	A high and mutual commitment to the relationship was positively related to greater adjustment.
[89]	1311	NR, 20.5 (NR)	Body satisfaction, self-esteem, depressive symptoms, and suicidal ideation.	Scores of well-being were generally consistent across sex partner categories (stranger, casual, close, exclusive, spouse, other), and no significant associations between partner type and well-being were found.
[33]	235	18–27, 21.73 (1.64)	Life satisfaction and positive and negative affect.	Relationship quality and need satisfaction were directly and indirectly related to well-being.
[49]	567	15–19, 16.1 (1.01)	Psychological deterioration.	Psychological deterioration was one of the most common consequences of violence in dating relationships.
[34]	Married: 65 couples Dating: 66 couples	Married: NR, 28.39 (7.05) Dating: NR, 21.49 (2.14)	Relational satisfaction and stability.	A greater breadth of positive relationship experiences was concurrently and longitudinally associated with well-being.
[35]	1500	15–25, 21.50 (2.99)	Expressions of love and support, communication, and perceived risk of negative relationship outcomes.	Congruence between relationship ideals and experiences was positively associated to well-being.
[90]	58 couples	Males: NR, 22 (NR) Females: NR, 21 (NR)	Relationship satisfaction.	Paying more attention to positive partner behaviors rather than negative partner behaviors was positively associated to well-being.
[60]	30	18–25, 23.4 (NR)	Level of happiness.	Romantic involvement was associated to a positive quality of life, positive feelings of happiness, and reducing negative states such as anger and sadness.
[36]	1582	North America: 18–54, 19 (0.13) Africa: 17–45, 25.18 (0.23) Europe: 17–66, 23 (0.35)	Life satisfaction with life, positive and negative affect, and personal satisfaction.	Attachment security was the main predictor of well-being in the American and European samples, while in the Mozambican samples it was the Eros love style. Attachment security and well-being was not gender-specific.
[91]	61 couples	16–20, NR (NR)	Depressive symptomatology and self-esteem.	Romantic relationships characterized by inequality in the contribution of emotional resources and in decision-making, were associated with greater psychological symptomatology.
[92]	105 couples	17–26, 19.2 (1.8)	Life satisfaction and positive and negative affect.	Higher goal conflict was directly associated with lower relationship quality and lower well-being.
[93] Study 2	56	Male: 18–22, 19.3 (1.3) Female: 18–20, 18.5 (0.6)	Happiness, anger, worry, and sadness.	Engaging in goal-congruent activities with a partner was associated with the highest reports of well-being.
[94] Study 1	187	19–54, 21.51 (3.35)	Daily life satisfaction.	Single people high in avoidance goals were just as happy as people involved in a relationship. In addition, individuals high in approach goals experienced greater well-being, but particularly when they were involved in a relationship.
[95]	92	18-27, 20.34 (2.28)	Perceived impact of body feedback.	Positive messages from partners about the own body increased confidence, self-acceptance, and sexual empowerment/fulfillment, whereas negative messages decreased these feelings.
[96]	130 couples	Males: NR, 26.5 (4.2) Females: NR, 25.4 (3.5)	Marital satisfaction.	High levels of positive affect in conflict situations were positively associated with relationship satisfaction and stability.
[97]	37,855	NR, 29.8 (4.4)	Life satisfaction and positive affect.	Divorce predicted higher well-being when initial relationship quality was poor.
[98]	Sample 1: 78 couples Sample 2: 132 couples	Sample 1: 21–55, 25 (5.9) Sample 2: 18–67, 24.2 (5.8)	Psychological need fulfillment (relatedness, autonomy and competence—self-determination theory).	Anxious and avoidant attachment predicted lower well-being.
[99]	68	18–36, 25.52 (3.74)	Depression, life satisfaction, and perceived stress.	Communal coping was unrelated to psychological distress. Partner overinvolvement in diabetes management had a mixed relation to outcomes, whereas partner under involvement was uniformly related to poor outcomes.
[100]	387	14–17, 15.47 (1.05) -at enrolment-	Relationship quality, partner meets needs, fertility control attitudes, condom use efficacy, sexual negativity, sexual satisfaction, absence of genital pain, partner sexual communication, closeness to family, partner’s closeness to family, general communication with family, substance use, smoking, depression, thrill seeking, self-esteem, anticrime attitudes, anti-deviance attitudes, peer substance use, religiosity, attitudes toward education, community group membership, school group membership, and volunteer work.	Higher sexual health was significantly associated with less substance use, lower self-reported depression, lower thrill seeking, higher self-esteem, having fewer friends who use substances, higher religiosity, better social integration, a lower frequency of delinquent behavior and crime, and more frequent community group membership.
[101]	30 couples	18–25, 19.4 (NR)	Relationship quality satisfaction.	Correspondence between personal and normative scripts, and agreement between partners on personal scripts predicted well-being.
[102] Study 1a	99	NR, 18.72 (1.02)	Relationship valuation.	As participants’ chronic promotion concerns increased, the association between autonomy support and relationship valuation was stronger.
[102] Study 1b	112	NR, 27.78 (9.49)	Commitment and relationship satisfaction.	The perceived support of one’s autonomy needs within a romantic relationship was positively associated with well-being.
[102] Study 3a	87 couples	NR, 20.55 (2.03)	Relationship quality.	Support for autonomy was judged more relevant among individuals concerned with promotion, while support for relatedness would be judged more relevant among individuals concerned with prevention.
[103] Study 2	153	18–38, 20.1 (2.4)	Positive and negative affect, life satisfaction, relationship satisfaction, quality, conflict, and commitment.	Approach motives for sacrifice were positively associated with well-being and relationship quality, while avoidance motives for sacrifice were negatively associated with well-being.
[104] Part 2	80 couples	18–60, 23.9 (6.4)	Positive and negative emotions and life satisfaction.	Within-person increases in emotional suppression during daily sacrifice were associated with decreases in well-being.
[105]	124	18–38, 20.2 (2.6)	Positive and negative affect, life satisfaction, relationship satisfaction, closeness, fun, and conflict.	Approach sex motives were positively associated with well-being, while avoidance sex motives were negatively associated.
[106]	295	Males: 18–21, 19.25 (NR) Females: 18–21, 19.19 (NR)	Self-esteem, loneliness, social anxiety, and avoidance.	Romantic relationship intimacy was positively associated to well-being
[46]	691	NR, 23 (NR)	Relationship happiness, life satisfaction, general happiness, distress symptoms, and self-esteem.	Individuals in happy relationships reported a higher level of well-being than did individuals in unhappy relationships. Married individuals reported the highest level of well-being, followed cohabiting, steady dating, and casual dating.
[107]	184	At age ≈ 14: 14.26 (0.76) At age ≈ 15: 15.21 (0.81) At age ≈ 25: 25.67 (0.96)	Positive and negative affect.	Early adolescent positive affect predicted fewer relationship problems and healthy adjustment to adulthood.
[108]	193	17–23, 19.16 (1.20)	Relationship satisfaction and commitment.	Sexual compliance was negatively associated with well-being.
[109]	113	In-relationship group: NR, 21.8 (0.3) No-relationship group: NR, 21 (0.2)	Subjective happiness.	Being in a romantic relationship was associated with reduced gray matter density in striatum and increased subjective happiness.
[110] Study 1	62 couples	18–37, 21.52 (3.51)	Positive and negative affect and life satisfaction.	Autonomy support between romantic partners was significantly positively related to goal progress. The beneficial effect of autonomy support was mediated by enhanced autonomous goal motivation.
[110] Study 3	426	18–58, 26.50 (7.53)		Autonomy support similarly promoted progress at vicarious goals.
[111]	231 couples	NR, 27.10 (NR) -time 4-	Dyadic adjustment.	Locomotion was positively associated with partner affirmation, movement toward the ideal self, and well-being.
[112]	51	21–29, 27.02 (1.88)	Depressive symptoms and life satisfaction.	Relationship quality and forming subsequent romantic relationships after breakup did not predict the changes in well-being, whereas remaining single after a breakup was negatively associated with depressive symptoms.
[113]	73	17–29, 19.5 (2.3)	Positive and negative emotions and life.	Higher levels of interdependence increased well-being if partners suppressed their negative emotions during sacrifice.
[114]	209	NR, 19.6 (1.6)	Psychological distress, alienation, life satisfaction, self-esteem, and psychological maturity.	Self-determination and a secure attachment style were both positively associated to well-being.
[115] Study1	362	18–57, 22.87 (7.47)	Physical and psychological symptoms and relationship commitment.	Greater romantic secrecy was associated with reduced commitment to relationship and more reported health symptoms.
[115] Study2	368	18–59, 22.47 (6.98)	Physical and psychological symptoms and relationship commitment.	Romantic secrecy was negatively associated with relational commitment and positively related to negative affect.
[116]	32,479	11–16, 13.6 (1.4)	Life satisfaction.	Experiences of dating violence were associated with poorer well-being.
[117]	203 couples	NR, 22.69 (5.49)	Positive and negative affect and life satisfaction.	Partner perfectionist concerns were negatively associated to well-being.
[118]	100	NR, 26.4 (0.86)	Happiness and positive and negative affect.	High levels of intimacy were positively associated to well-being.
[119]	20,000 (4 cohorts)	C1-T1: 18; T10: 33 C2-T1: 17; T10: 30 C3-T1: 16; T9: 24 C4-T1: 17; T4: 20	Life satisfaction.	Marriage and de facto relationships were positively associated to well-being.
[120] Study 1	473	NR, 19.96 (2.81)	Depressive symptoms.	Experiencing situations of physical or psychological abuse was associated with lower levels of well-being.
[121]	277	NR, 29.79 (6.54)	Depressive symptoms, relationship satisfaction, and sexual functioning.	Communication had a beneficial effect on both the individual and the dyadic level in the context of existence of sexual problems.
[52]	12,504	7th –12th grades	Depressive symptoms.	Interracial daters had greater odds of risk for depression than their non-dating and same-race dating peers. Experiencing a romantic breakup explained the elevated risk of depression for daters in general, and same-race daters specifically, but not interracial daters.
[122]	1239	13–18, 15 (1.63)	Life satisfaction.	A significant, although weak interaction effect of stress related to romantic relationships by sense of coherence was found in association with life satisfaction for boys. The other interaction effects were nonsignificant in both genders
[123]	461	17–21, 18.90 (1.14)	Loneliness, academic satisfaction, and stress.	A secure attachment style was positively associated with well-being.
[124]	121 couples	NR, 19.5 (NR)	Relationship satisfaction, ambivalence, and conflict.	Partner idealization was positively associated with well-being.
[125]	314	European Americans: 18–59, 26.8 (10.5) Mexican Americans: 17–55, 26.1 (7.8)	Relational self-esteem and depression.	Power inequality was associated with a lack of authentic self-expression in both populations. A lack of authenticity negatively impacted psychological health, especially for Mexican Americans.
[126]	264 couples	Males: NR, 27 (NR) Females: NR, 25 (NR)	Happiness with the marriage, satisfaction with the marriage, happiness with the level of equity in the marriage, perceived stability of the marriage, perceived certainty that they would still be married in 5 years, and frequency of thoughts of leaving the spouse.	Reporting abundant and positive experiences and giving positive meaning to them were associated with improved levels of well-being over time.
[127]	832	17–54, 20 (2.85)	Psychological distress.	Young adults who reported negative and ambivalent emotional reactions to hooking up also reported lower well-being.
[128]	122	Victims: NR, 19.2 (NR) Nonvictims: NR, 19 (NR)	Psychological distress.	Psychological distress was not significantly predicted by coping strategies or the interaction of control and coping in situations of relationship violence.
[129]	256	19–28, 23 (2.55)	Life satisfaction with life and positive and negative affect.	The maintenance of relational behaviors driven by self-determined motives was positively associated to well-being.
[130]	161	Undergraduate, 17–66, NR (NR)	Depression symptoms, life satisfaction, satisfaction with oneself, and physical health.	Having a romantic relationship was associated significantly with well-being, however, results showed that they may be detrimental to women’s well-being
[131]	176	NR, 20.94 (3.07)	Somatization, depression, anxiety and self-esteem.	Male gender roles, such as success, competitiveness, or power, were negatively associated with the well-being of partners.
[132]	255 couples	Males: 20–45, 28.93 (4.05) Females: 20–45, 27.20 (3.31)	Relationship adjustment, sexual satisfaction, and sexual desire.	Dyadic empathy was positively associated to well-being.
[133] Study 2	400	18–26, 19.62 (1.95)	Psychiatric disorders.	Low relationship quality levels were negatively associated to well-being.
[134] Study 1	187 couples	NR, 24.97 (4.62) -time 1-	Dyadic adjustment.	Partner similarity was positively associated to well-being.
[134] Study 2	137 couples	NR, 26.45 (4.56) -time 3-		
[135]	58	18–23, 18.8 (1.1)	Religious well-being and existential well-being.	Forgiveness was associated with greater well-being.
[136]	50 couples	18–70, 22.75 (10.60)	Depression, life satisfaction, empathic concern, and relationship satisfaction.	Emotional interdependence between partners was positively related to well-being, especially regarding positive emotions.
[137]	176	24–29, 24.13 (1.84)	Depression and anxiety.	Higher levels of anxiety and depressive symptoms predicted increases in negative romantic experiences.
[37]	5316	Boys: NR, 16.06 (1.51) Girls: NR, 15.76 (1.48)	Severe depression, suicidal ideation, and suicide attempt.	Romantic relationship inauthenticity was positively associated with the risk of depression, suicide ideation and attempt, but only for girls.
[138]	2818	18–30, 24 (3.86) -waves 1 and 3-	Life satisfaction.	Relationship status was related to well-being, reporting married young adults the highest level.
[139]	110	13–18, 16.7 (NR)	Depression, anxiety, self-esteem, mastery, and life satisfaction.	A high-quality relationship was associated with increased self-esteem.
[140]	4564	11–21, 16.16 (1.51)	Depression and anxiety.	Interracial daters experienced more symptoms of depression and anxiety and poorer family relationships than same-race daters.
[38]	100 (time 4)	Age 29 -time 4-	Depression and anxiety.	Romantic relationships turning points were related to well-being. A negative turning point was associated to greater depressive symptoms. A positive turning point or a formal turning point were associated to more healthy romantic relationships and a lower number of symptoms.
[141]	11,695	18–28, 21.82 (1.85)	Life satisfaction.	Married young adults reported higher life satisfaction than those in other type of romantic relationships, those in no romantic relationship, and those who married prior to age 22.
[142]	466	16.22, 17.82 (0.92)	Depression symptoms and self-esteem.	Dating violence victimization was linked with symptoms of depression and a lower self-esteem.
[39]	3258	15–21, NR (NR)	Self-esteem, depression, isolation, verbal aggression, delinquent behaviors, benevolent sexism, and hostile sexism.	Adolescents who had a very good-quality relationship reported higher levels of psychological adjustment.
[143] Study 1	127 couples	NR, 23.33 (3.65)	Life satisfaction, stress, and relationship satisfaction.	Self-control significantly predicted higher life satisfaction and lower stress. However, relationship satisfaction was not significantly predicted by self-control.
[143] Study 2	149 couples	NR, 25.83 (4.41)	Life satisfaction, subjective well-being, psychological and dyadic adjustment.	Self-control predicted higher life satisfaction, well-being, psychological adjustment, dyadic adjustment, and relationship satisfaction.
[144]	666	18–24, NR (NR)	Depression, anxiety, life satisfaction and self-esteem.	Hook-ups were associated with higher well-being for women and lower well-being for men.
[145]	119	NR, 23 (2.28)	Autonomy, environmental mastery, personal growth, purpose in life, positive relationships, and self-acceptance (Ryff’s model of psychological well-being).	Positive relationship quality was found to be a mediator between forgiveness (seeking and self) and well-being.
[146]	145	18–25, 21.10 (1.75)	Happiness, psychological distress, and self-esteem.	Low attachment anxiety in romantic relationships predicted happiness; low attachment anxiety and high self-efficacy predicted low psychological distress; less fear of negative evaluation from the partner and high self-efficacy positively predicted self-esteem.
[147]	484	18–25, 19.13 (1.47)	Depressive symptoms.	Higher relationship quality was positively associated with well-being.
[148] Study 2	60	NR, 19.7 (2.78)	Depressive symptoms.	Self-blame predicted depressive affect to the extent that participants forgave themselves.
[149]	506	17–24, 20.79 (1.24)	Relational self-esteem and relational depression.	Higher levels of self-compassion were related to greater likelihood to compromise, as well as greater authenticity, lower levels of emotional turmoil, and higher levels of well-being.
[150]	31	21–24, 22.1 (0.98)	General affect and life satisfaction.	Forgiveness was positively related to improvement in anxiety, depression, and well-being.
[40]	148 couples	17–29, 20.8 (3.8)	Anxiety, depressed mood, positive well-being, self-control, general health, and vitality.	Individuals with better well-being reported more positive romantic behaviors.

Note: NR = information not reported.

**Table 2:** Categories, specific romantic variables, and measurement constructs of the included studies.

Category	Variables (Number of Studies)		Measurement Constructs	Included Studies (Reference)
**Relational Variables**	Relationship status (17)		Singlehood; relationship status (single/married/engaged/cohabiting/divorced, dating steadily/dating multiple people, etc.).	[44,46,62,63,74,75,81,82,89,94,97,112,119,127,138,141,144]
	Relationship quality (15)		Relationship adjustment; intimacy; communication; expectations about the future; conflicts; companionship; intimacy; reliable alliance; affection; relationship satisfaction; commitment; trust; passion; love; social support; depth; conflict; relationship happiness; acceptance; understanding; dyadic adjustment; positive and negative partner behaviors.	[13,22,33,39,40,46,60,85,92,97,112,133,139,145,147]
	Relationship history and experiences (12)		Relationship development; oral history coding; gratitude; daily interactions with partner; perception of partner responsiveness to gratitude expression; reminiscing about laughter; positive and negative events; relationship power; abuse; conflicts or disagreements; courtship story; relationship violence; negative romantic experiences; turning points.	[34,38,64,65,67,73,91,120,125,126,128,137]
	Commitment and intimacy (7)		Expression of commitment; mutuality; level of commitment; relationship status; long term orientation; feelings of psychological attachment; closeness; security; care; understanding; perceptions of positive intimacy; intimacy frequency and intensity; sexual intimacy; intimacy narrative.	[46,61,84,88,106,115,118]
	Romantic attachment (7)		Romantic attachment style.	[36,40,80,98,114,123,146]
	Communication and conflict resolution (5)		Partner messages; reciprocity; affect; disclosure of sexual problems; dyadic empathy; self-compassion.	[95,96,121,132,149]
	Need fulfillment (7)		Autonomy, competence and relatedness satisfaction (SDT); social support.	[33,77,98,102,114,129,130]
	Relational and personal goals (6)		Michelangelo phenomenon; goal progress; goal congruence; goal support.	[87,92,93,110,111,134]
	Dating violence (7)		Physical/verbal/sexual/psychological/ emotional victimization; physical/emotional/verbal aggression; rape.	[47,48,49,78,83,116,142]
	Sexual minority youth (3)		Same sex relationships.	[68,72,86]
	Interracial relationships (2)		Interracial daters; same-sex daters; non-daters.	[52,140]
	Sexuality (2)		Sexual activity; sexual health.	[45,100]
	Others (7)	Emotional interdependence	Daily interactions; mood; partner support; daily emotions.	[90,99,136]
		Shared efficacy	Relationship efficacy of dyad.	[66]
		Partner perfectionistic concerns	Socially prescribed perfectionism; concern over mistakes; self-criticism.	[117]
		Neurological effect	Striatum gray matter density.	[109]
Personal Variables	Romantic relationship inauthenticity (1)		Ideal romantic relationship events vs. actual events.	[37]
	Sense Of Coherence (1)		Comprehensibility; manageability; meaningfulness.	[122]
	Positive and negative affect (1)		Affective arousal.	[107]
	Relationship expectations and believes (4)		Relationship scripts; marriage myths; benevolent sexism; positive illusions.	[32,35,101,124]
	Behaviors (9)		Negative maintenance behaviors; authenticity; theory of mind; sexual compliance; self-control.	[59,69,76,79,108,113,125,143,149]
	Motivation (8)		Forgiveness; approach and avoidance motives; emotional suppression and expression; sacrifice.	[79,103,104,105,113,135,148,150]
	Coping (4)		Explicit attitudes towards partner; mindfulness; coping strategies.	[70,71,120,128]
	Others (4)	Reactance	Attitude toward infidelity.	[31]
		Gender role conflict	Men’s thoughts and feelings concerning gender role behaviors.	[131]
		Romantic Competence	Insight; mutuality; emotion regulation.	[43]
		Abilities	Self-efficacy; relational anxiety.	[146]
